# The Seneca Valley virus 3C protease cleaves DCP1A to attenuate its antiviral effects

**DOI:** 10.1186/s13567-025-01477-0

**Published:** 2025-02-28

**Authors:** Jingjing Yang, Zijian Li, Ruiyi Ma, Shijie Xie, Dan Wang, Rong Quan, Xuexia Wen, Jiangwei Song

**Affiliations:** 1 Key Laboratory of Livestock Infectious Diseases, Ministry of Education, and Key Laboratory of Ruminant Infectious Disease Prevention and Control (East), Ministry of Agriculture and Rural Affairs, College of Animal Science and Veterinary Medicine, Shenyang Agricultural University, 120 Dongling Road, Shenyang, 110866 China; 2 Beijing Key Laboratory for Prevention and Control of Infectious Diseases in Livestock and Poultry, Institute of Animal Husbandry and Veterinary Medicine, Beijing Academy of Agriculture and Forestry Sciences, No. 9 Shuguang Garden Middle Road, Haidian District, Beijing, 100097 China

**Keywords:** Seneca Valley virus (SVV), DCP1A, 3C protease, 3D, cleavage

## Abstract

Seneca Valley virus (SVV), a new member of *Picornaviridae*, causes idiopathic vesicular symptoms in pregnant sows and acute death in neonatal piglets, considerably damaging the swine industry. The viral protease 3C (3C^pro^) cleaves host immune-related molecules to create a favorable environment for viral replication. In this study, we found that mRNA decapping enzyme 1A (DCP1A) is a novel antiviral effector against SVV infection that targets 3D viral RNA-dependent RNA polymerase for OPTN-mediated autophagic degradation. To counteract this effect, SVV 3C^pro^ targets DCP1A for cleavage at glutamine 343 (Q343), resulting in the cleaved products DCP1A (1–343) and DCP1A (344–580), which lose the ability to restrict SVV replication. In contrast, the 3C cleavage-resistant DCP1A-Q343A mutant exhibited stronger antiviral effects than the wild-type DCP1A. Additionally, the degradation of the viral 3D protein targeted by DCP1A was abolished after its cleavage by SVV 3C^pro^. In conclusion, our study demonstrated that SVV 3C^pro^ is a pivotal ISG antagonist that cleaves DCP1A. These results offer novel insight into how viruses evade host immunity.

## Introduction

Seneca Valley virus (SVV) is the only member of the genus *Senecavirus* (family *Picornaviridae*) and a single serotype [1]. It was initially identified as a contaminant in the cell culture medium during PER. C6 cell propagation and transformation into fetal retinoblasts. Although SVV is not pathogenic to normal human cells, it has significant oncolytic activity against cancer cells, including neuroendocrine tumors and human retinoblastoma cells [2, 3]. Furthermore, SVV causes vesicular diseases and transient epidemic neonatal losses in swine. The first outbreak of SVV infection in China occurred in 2015 in Guangdong Province and manifested as the sudden death of newborn piglets across multiple farms [4]. Since then, SVVs have progressively spread to other Chinese provinces.

SVV is a nonenveloped virus with linear, nonsegmented, single-stranded, positive-sense strand RNA [5]. The genome is approximately 7.3 kb in length and has a 3′ poly (A) tail but lacks a 5′ cap structure. It encompasses 5′ and 3′ untranslated regions (UTRs), as well as a single long open reading frame (ORF) of the polyprotein precursor. The 5′UTR harbors a type IV internal ribosome entry site, enabling translation initiation in a cap-independent manner [6]. The 3′UTR contains a kissing loop structure, followed by a poly (A) sequence. Like other picornaviruses, the polyprotein precursor of SVV is stepwise cleaved into 12 polypeptides with a layout of “leader sequence—four P1 polypeptides—three P2 polypeptides—four P3 polypeptides” [7]. The 3C protease processes P1 polypeptides into VP0, VP3, and VP1; VP0 is then further cleaved into VP4 and VP2. VP1-4 are structural proteins that encapsulate the SVV genome. Similarly, the P2 and P3 regions are processed into seven nonstructural proteins: 2A, 2B, 2C, 3A, 3B (VPg), 3C protease, and 3D polymerase. The primary cleavage event involves a ribosomal skipping mechanism and occurs at the conserved TNPG↓P motif between 2A and 2B (the arrow indicates the cleavage site).

The interferon (IFN) and IFN-induced Janus kinase signal transducer and activator of transcription (JAK-STAT) signaling pathways are the first lines of defence against viral invasion. They inhibit viral infection through the upregulation of hundreds of interferon-stimulated genes (ISGs) [8]. Processing bodies (P-bodies) are crucial sites for mRNA decapping and degradation in mammalian eukaryotic cells [9]. They express mRNA-decapping enzyme 1A (DCP1A), which removes the 5′ N7-methylguanosine cap to induce mRNA degradation [10]. As an ISG, DCP1A has demonstrated antiviral activity against multiple viruses [11, 12].

Viruses have developed strategies to counteract host ISGs. Anti-ISG adaptations include inhibiting transcription [13], downregulating ISG protein expression [14], altering subcellular localization [15], and blocking ISG protein expression via direct interaction [16]. A number of studies have elucidated the mechanisms by which viruses antagonize the antiviral activity of DCP1A. For example, the EVH1 domain of DCP1A interacts with PKR to induce eIF2α phosphorylation and inhibit protein translation in poliovirus [17]. In response, poliovirus employs 3C^pro^ to cleave and degrade DCP1A [18]. Nonstructural protein 5 (nsp5) of porcine deltacoronavirus (PDCoV) is a 3C-like protease that cleaves DCP1A at residue Q343 [11]. Both alphacoronaviruses (e.g., porcine epidemic diarrhea virus [PEDV], human coronavirus [HCoV]-229E, HCoV-NL63, swine acute diarrhea syndrome coronavirus [SADS-CoV]) and betacoronaviruses (HCoV-OC43, HCoV-HKU1, severe acute respiratory syndrome coronavirus [SARS-CoV], SARS-CoV-2, middle east respiratory syndrome coronavirus [MERS-CoV]) have nsp5 that performs the same cleavage function [11, 19, 20]. In SADS-CoV, for example, nsp5 cleaves DCP1A to inhibit the IRF3 and NF-κB signaling pathways, thus blocking the host inflammatory response [19]. The porcine reproductive and respiratory syndrome virus 3C-like protease nsp4 is also capable of cleaving porcine DCP1A, but it targets residue E238 instead of residue Q348, the target of coronaviruses [21]. The 3C protein encoded by SVV is also a 3C-like protease, and whether it has similar effects on DCP1A as proteases from other viruses is unknown.

In this study, we identified porcine DCP1A as an anti-SVV infection factor that targets 3D viral RNA-dependent RNA polymerase for autophagic degradation. SVV infection led to the degradation and cleavage of DCPlA, and viral 3C^pro^ cleaved DCP1A to abrogate its antiviral effect. Our results revealed the mechanism underlying the immune evasion strategy employed by SVV.

## Materials and methods

### Cells, viruses, antibodies, and reagents

BHK-21 cells (CCL-10), HEK-293T cells (CRL-11268) and PK-15 cells (CCL-33) were cultured in Dulbecco’s modified Eagle’s medium (DMEM; Invitrogen, Madison, WI, USA) supplemented with 10% fetal bovine serum (FBS; Invitrogen), 100 U/mL penicillin, and 100 μg/mL streptomycin at 37 °C in 5% CO_2_. The SVV strain CHhb17 (GenBank: MG983756.1) was propagated in BHK-21 cells [22]. The eGFP-inserted rescued Seneca Valley virus (rSVV-eGFP) was graciously provided by Dr. Fuxiao Liu (Qingdao Agricultural University) [23]. The SVV VP1 mouse monoclonal antibody used in this study was previously described [22]. Rabbit anti-HA monoclonal antibody (3724) was purchased from Cell Signaling Technology (Beverly, MA, USA), mouse anti-HA monoclonal antibody (A1933) from ABclonal (Wuhan, China), and mouse anti-myc monoclonal antibody (M4439) from Sigma-Aldrich (St. Louis, MO, USA). GFP mouse monoclonal antibody (ab127417), β-actin mouse monoclonal antibody (ab8226) and rabbit anti-myc antibody were purchased from Abcam (Cambridge, MA, USA). GFP rabbit polyclonal antibody (50430-2-AP) and rabbit DCP1A polyclonal antibody (22373-1-AP) were purchased from Proteintech (Wuhan, China). Alexa-568-conjugated goat anti-rabbit secondary antibody (A11011), Alexa-488-conjugated goat anti-mouse secondary antibody (A11001) and Alexa-647-conjugated goat anti-mouse secondary antibody (A32728) were obtained from Invitrogen. HRP-conjugated goat anti-rabbit IgG (H + L) (1706515) and HRP-conjugated goat anti-mouse IgG (H + L) (1706516) were obtained from Bio-Rad (Hercules, CA, USA). MG132 (proteasome inhibitor, S2619), Z-VAD-FMK (caspase inhibitor, S7023), bafilomycin A1 (autophagy inhibitor, Baf A1, S1413), chloroquine (autophagy inhibitor, CQ, S6999), and 3-methyladenine (autophagy inhibitor, 3-MA, S2767) were purchased from Selleck Chemicals (Shanghai, China). Ammonium chloride (NH_4_Cl, HY-Y1269) was purchased from MedChemExpress (Shanghai, China).

### Plasmid construction

GFP-tagged SVV structural and nonstructural protein expression plasmids and GFP-3C mutations (H48A or C160A), as well as double mutations (H48A-C160A), HA-SQSTM1/p62, and HA-OPTN, which were employed in our previous studies [22, 24, 25], were used. The pig DCP1A gene (GenBank: NM_001244358.1) was amplified from the cDNA of PK-15 cells and cloned and inserted into the pCMV-myc vector. HA-tagged pig Tollip, NDP52, and NBR1 were synthesized by RuiBiotech Biotechnology Co., Ltd. (Beijing, China). The truncated mutants DCP1A were also cloned and inserted into the pCMV-myc vector. Site-directed mutagenesis was performed to generate four mutants of DCP1A with KOD DNA polymerase (KFX-201; TOYOBO, Osaka, Japan), including DCP1A (E325A), DCP1A (Q330A), DCP1A (Q343A), and DCP1A (Q351A). The primers used are listed in Table 1.

**Table 1:** **Primers used in this study**

Primers^a^	Sequence (5′-3′)^b^
myc-DCP1A-EcoRI-F myc-DCP1A-KpnI-R myc-DCPIA (1–343)-KpnI-R myc-DCPIA (344–580)-EcoRI-F myc-DCPIA (1–320)-KpnI-R myc-DCPIA (1–220)-KpnI-R myc-DCPIA (E325A)-F myc-DCPIA (E325A)-R myc-DCPIA (Q330A)-F myc-DCPIA (Q330A)-R myc-DCPIA (Q343A)-F myc-DCPIA (Q343A)-R myc-DCPIA (Q351A)-F myc-DCPIA (Q351A)-R qRT-PCR-DCP1A-F qRT-PCR-DCP1A-R qRT-PCR-GADPH-F qRT-PCR-GADPH-R	TGGCCATGGAGGCCCGAATTCGGATGGAGTCGCTGAGTCGAGCTG GATCCCCGCGGCCGCGGTACCTCATAGGTTGTGGTTGTCTTTG GATCCCCGCGGCCGCGGTACCCTGCATCATGGTGGTGCTTCGG TGGCCATGGAGGCCCGAATTCGGGCAGTGAAGACCACGCCTAGAC GATCCCCGCGGCCGCGGTACCGGGACTGAGCACAGGGTGCAAC GATCCCCGCGGCCGCGGTACCTTCCACCGTCAGATGTTTGTGT ACCAGCAGCGGCTTCTACTGCACAGGCTCCTC TAGAAGCCGCTGCTGGTAGAGTGGGACTGAGC AAGCTTCTACTGCAGCGGCTCCTCCCAGCTTACCCC CGCTGCAGTAGAAGCTTCTGCTGGTAGAGTGG ATGATGGCGGCAGTGAAGACCACGCCTAGACA TTCACTGCCGCCATCATGGTGGTGCTTCGGGG CTAGAGCGAGGTCTCCACTCTCGAGTCAGCCA TGGAGACCTCGCTCTAGGCGTGGTCTTCACTGC GGAATTTCAGCTCCATGAACCA TTGGCCTGGCTGGGACTCTGTT GAGTCAACGGATTTGGTCGT GACAAGCTTCCCGTTCTCAG

^a^F denotes forward PCR primer; R denotes reverse PCR primer.

^b^Restriction sites are underlined.

### Coimmunoprecipitation and western blotting

For coimmunoprecipitation (co-IP), BHK-21 cells in 6-well plates were cotransfected with plasmids with Lipofectamine 2000 reagent (BL623B; Biosharp, Hefei, Anhui, China). At 24 h post-transfection (hpt), the cells were lysed with lysis buffer (P0013J; Beyotime, Shanghai, China) and rotated at 4 °C on ice for 30 min. The cell lysates were centrifuged at 13 000 rpm for 15 min, after which the cellular debris was removed. A portion of the supernatant was kept as a control and heated directly, while the rest was mixed with anti-myc magnetic beads (HY-K0206; MedChemExpress) and rotated overnight. The beads were washed with cell lysis solution, resuspended in 1 × SDS loading buffer and boiled at 100 °C. The subsequent steps were the same as those for western blotting.

For western blotting, the proteins were transferred onto a nitrocellulose membrane (66485; PALL, New York, NY, USA) after sodium dodecyl sulfate–polyacrylamide gel electrophoresis (SDS-PAGE). The membranes were then blocked with 5% skim milk (BS102; Biosharp) in phosphate-buffered saline (PBS). Anti-myc, anti-HA, and anti-GFP antibodies were used to detect the respective proteins. A rabbit DCP1A polyclonal antibody was used to detect the endogenous DCP1A protein during SVV infection. β-actin expression in each sample was used as a control. All analyses were performed via Image J software (National Institutes of Health, Bethesda, MD, USA).

### Quantitative reverse transcription PCR (qRT-PCR)

Total RNA was extracted via the FastPure Cell/Tissue Total RNA Isolation Kit (RC101-01; Vazyme, Nanjing, Jiangsu, China) following the manufacturer's instructions. RNA purity was determined via spectrophotometry and agarose gel electrophoresis. The RNA (1000 ng) was reverse transcribed with a first-strand synthesis master mix (F0202A; Lablead, Beijing, China). qRT-PCR was performed via a Bio-Rad CF96 Real-Time PCR system employing SYBR Green PCR mix (R0202A). We quantified the relative gene expression by calculating 2^−ΔΔCT^ (with CT standing for the threshold cycle). The primers used for the quantification of transcripts via qRT-PCR are shown in Table 1.

### Indirect immunofluorescence (IFA)

BHK-21 cells were transfected with the indicated plasmids for 24 h, fixed with 4% paraformaldehyde for 15 min, permeabilized, and blocked with 2% bovine serum albumin (BSA) and 0.1% Triton X-100. The cells were then incubated with the corresponding primary antibody or secondary antibody, followed by washing with PBS before the cell nuclei were stained with 4′,6-diamidino-2-phenylindole (DAPI) to remove residual antibodies. The images were captured via a Nikon Al confocal microscope (Tokyo, Japan).

### RNA interference (RNAi)

Small interfering RNAs (siRNAs) were designed and purchased from GenePharma (Suzhou, China). The siRNA sequences used were as follows: si-DCP1A (sense, 5′-CAAGCACUCAGCUCUCCAATT-3′; antisense, 5′- UUGGAGAGCUGAGUGCUUGTT-3′); si-OPTN (sense, 5′- GGACAAGAUGAUGCUGCAATT-3′; antisense, 5′-UUGCAGCAUCAUCUUGUCCTT-3′), siNC (sense, 5′-UUCUCCGAACGUGUCACGUTT-3′; antisense, 5′-ACGUGACACGUUCGGAGAATT-3′) used as a control. The cells were transfected with siRNAs using Lipofectamine RNAiMAX (13778150; Invitrogen) at a concentration of 20 pmol.

### TCID_50_ assay

BHK-21 cells were inoculated into 6-well cell culture plates. When the cells grew to form a monolayer of dense cells, they were infected with SVV at the indicated multiplicity of infection (MOI) and incubated at 37 °C with 5% CO₂ for 1 h. After being washed three times with DMEM, the medium was replaced with DMEM containing 2% FBS. The cells were collected at the indicated times, frozen and thawed three times, and titered via the TCID_50_ method.

### Statistical analysis

The data are shown as the means ± standard deviations (SDs) and were analyzed with GraphPad Prism software (GraphPad Software, San Diego, CA, USA). *P* values less than 0.05 were regarded as statistically significant.

## Results

### SVV infection cleaves and degrades DCP1A

**Figure 1:**
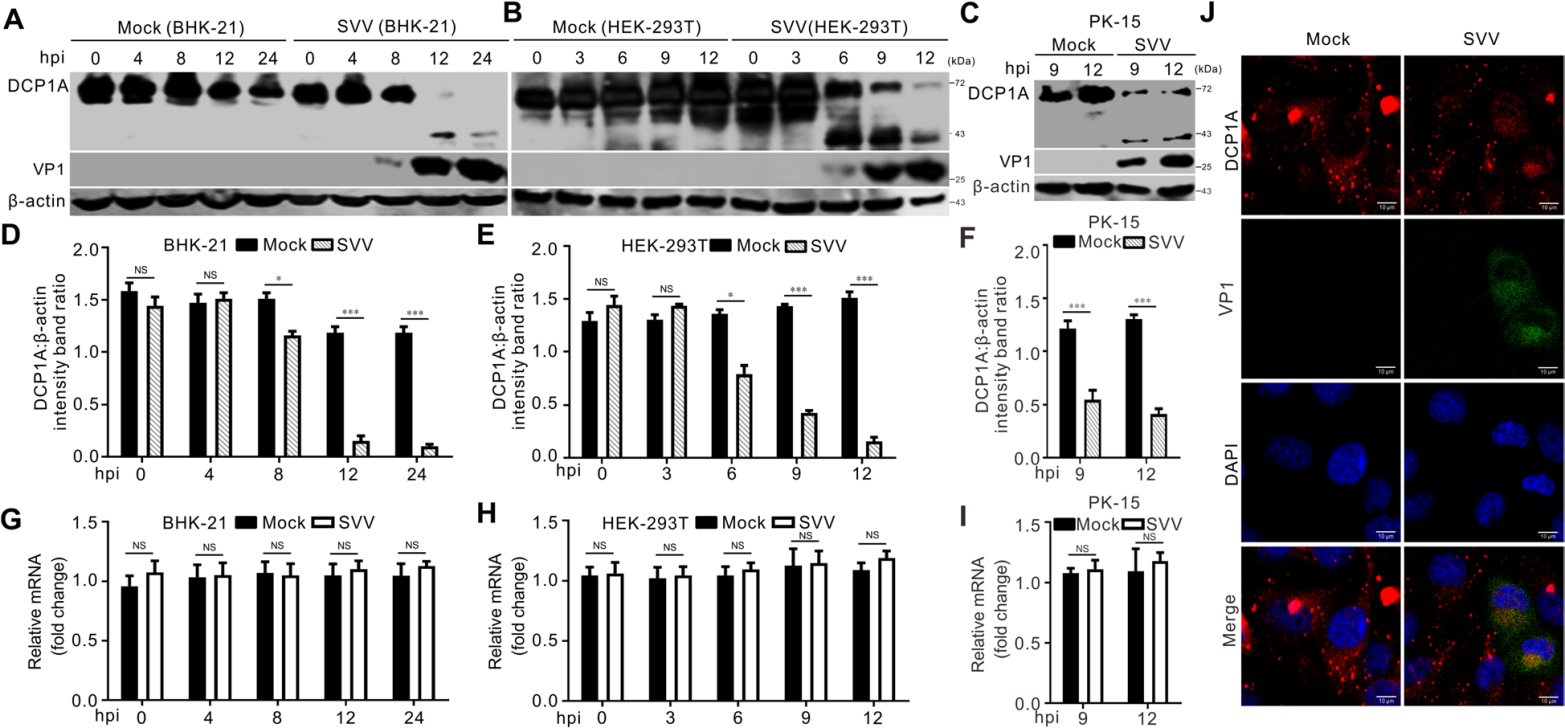
**SVV infection cleaves and degrades DCP1A. A–C** BHK-21 (**A**), HEK-293 T (**B**), and PK-15 (**C**) cells in 6-well plates were either mock infected or infected with SVV at an multiplicity of infection (MOI) of 5. The cells were harvested and subjected to western blotting analysis via antibodies against DCP1A, the VP1 protein, and β-actin at the indicated times post-infection. **D–F** Quantification of DCP1A expression in the images in (**A**–**C**) was conducted via Image J software. The intensity ratios of the DCP1A bands were normalized to those of β-actin. The data are presented as the means ± standard deviations (SDs) derived from three independent experiments. (NS, not significant; *P < 0.05; ***P < 0.001).** G–I** BHK-21 (**G**), HEK-293 T (**H**), and PK-15 (**I**) cells in 6-well plates were either mock infected or infected with SVV at an MOI of 5 for the indicated times. A quantitative real-time PCR (qRT-PCR) assay was performed to detect DCP1A mRNA levels. The mRNA levels of DCP1A were normalized to those of GAPDH. The data are presented as the means ± standard deviations (SDs) derived from three independent experiments. (NS, not significant). **J** BHK-21 cells in 6-well plates were either mock infected or infected with SVV at an MOI of 1. At 12 h post-infection (hpi), the cells were fixed, permeabilized, stained with antibodies against the DCP1A and VP1 proteins, and then examined via confocal microscopy.

To investigate whether SVV infection regulates DCP1A expression, we evaluated DCP1A protein levels in SVV-infected BHK-21, HEK-293 T, and PK-15 cells at different time points. As shown in Figures 1A–F, SVV infection resulted in a small cleavage band and significantly decreased DCP1A protein expression. In contrast, qRT-PCR assays indicated that SVV infection did not affect DCP1A mRNA levels (Figures 1G–I). Previous studies have shown that one of the mechanisms by which viruses counteract ISGs is by altering their normal subcellular localization [15]. To determine whether SVV infection affects the intracellular distribution of DCP1A, we examined the subcellular localization of DCP1A in SVV-infected BHK-21 cells. Confocal microscopy revealed that SVV infection disrupts the subcellular localization of DCP1A. The punctate distribution disappeared in SVV-infected cells, and a diffuse distribution was observed, which was not observed in mock-infected cells (Figure 1J). Hence, SVV infection induced DCP1A cleavage and degradation but did not affect its transcription.

### DCP1A inhibits SVV replication

**Figure 2:**
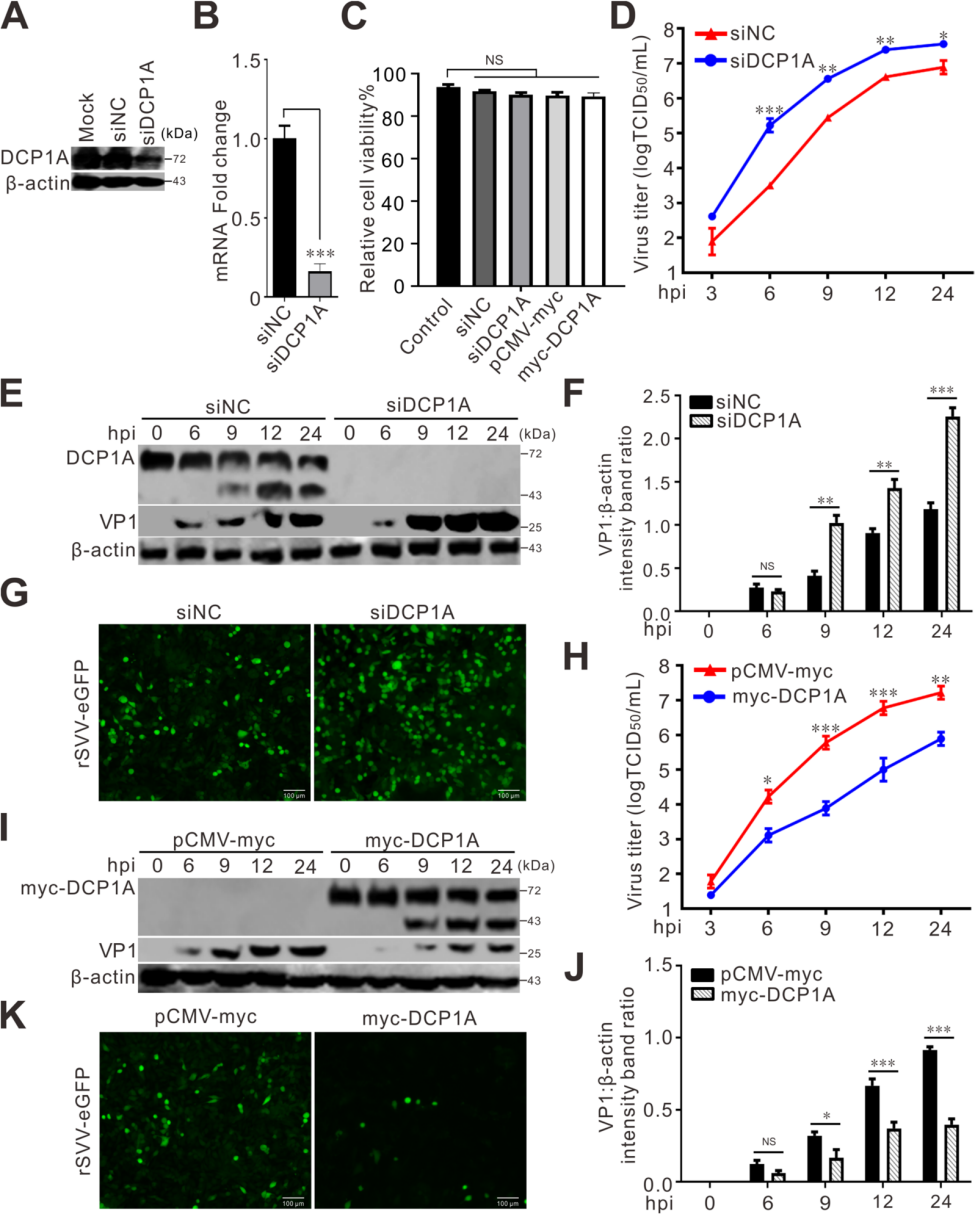
**DCP1A inhibits SVV replication. A**, **B** BHK-21 cells grown in 6-well plates were transfected with siRNAs targeting DCP1A (siDCP1A) or negative control (siNC) at a dose of 20 pmol. At 36 h post-transfection (hpt), western blotting (**A**) and qRT-PCR (**B**) were performed to examine the protein and mRNA levels of DCP1A, respectively. **C** A CCK-8 assay was used to assess the viability of BHK-21 cells transfected with siRNAs or plasmids. **D**, **E**, **H**, **I** BHK-21 cells plated in 6-well plates were first transfected with siRNAs (**D**, **E**) or 2 μg of plasmids (**H**, **I**), followed by infection with SVV at 36 hpt. TCID_50_ and western blotting assays were used to evaluate the viral titers in the supernatants (**D**, **H**) and the expression of the VP1 protein (**E**, **I**). **F**, **J** Quantification of VP1 expressions in **E** (**F**) and **I** (**J**) were conducted via Image J software. The intensity ratios of the VP1 bands were normalized to those of β-actin. The data are presented as the means ± standard deviations (SDs) derived from three independent experiments. (NS, not significant; *P < 0.05; **P < 0.01; ***, *P* < 0.001). **G**, **K** BHK-21 cells seeded in 6-well plates were transfected with siRNAs (**G**) or 2 μg of plasmids (**K**), followed by infection with rSVV-eGFP at 36 hpt. The fluorescence was visualized under a confocal microscope at 12 hpi.

We have shown that SVV infection cleaved DCP1A. Therefore, we investigated the role of DCP1A in SVV replication. First, we knocked down DCP1A via siRNA. The western blotting and qRT-PCR results revealed that DCP1A was significantly downregulated in BHK-21 cells transfected with siRNA targeting DCP1A (Figures 2A and B). As shown in Figure 2C, the results of the CCK-8 assay revealed that the transfection of siDCP1A did not affect BHK-21 cell viability. DCP1A knockdown increased viral titers and viral VP1 protein production at 6, 9, 12, and 24 h post-infection (hpi) (Figures 2D, E and F). The green fluorescence intensity from rSVV-eGFP in DCP1A-knockdown cells also increased at 12 hpi (Figure 2G). These results demonstrated that viral replication was notably enhanced. Second, we overexpressed DCP1A by transfecting the plasmid myc-DCP1A. As shown in Figure 2I, highly expressed myc-tagged DCP1A was detected via western blotting. The results of the CCK-8 assay revealed that plasmid transfection did not affect the viability of BHK-21 cells (Figure 2C). In contrast to DCP1A knockdown, the overexpression of DCP1A inhibited SVV replication. We drew this conclusion on the basis of a reduction in viral titer and VP1 protein in cells overexpressing DCP1A at 6, 9, 12 and 24 hpi, as well as low fluorescence intensity from rSVV-eGFP in DCP1A-overexpressing cells at 12 h hpi (Figures 2H–K). Overall, these results indicate that DCP1A is an antiviral factor against SVV infection.

### SVV 3C^pro^ cleaves DCP1A

**Figure 3:**
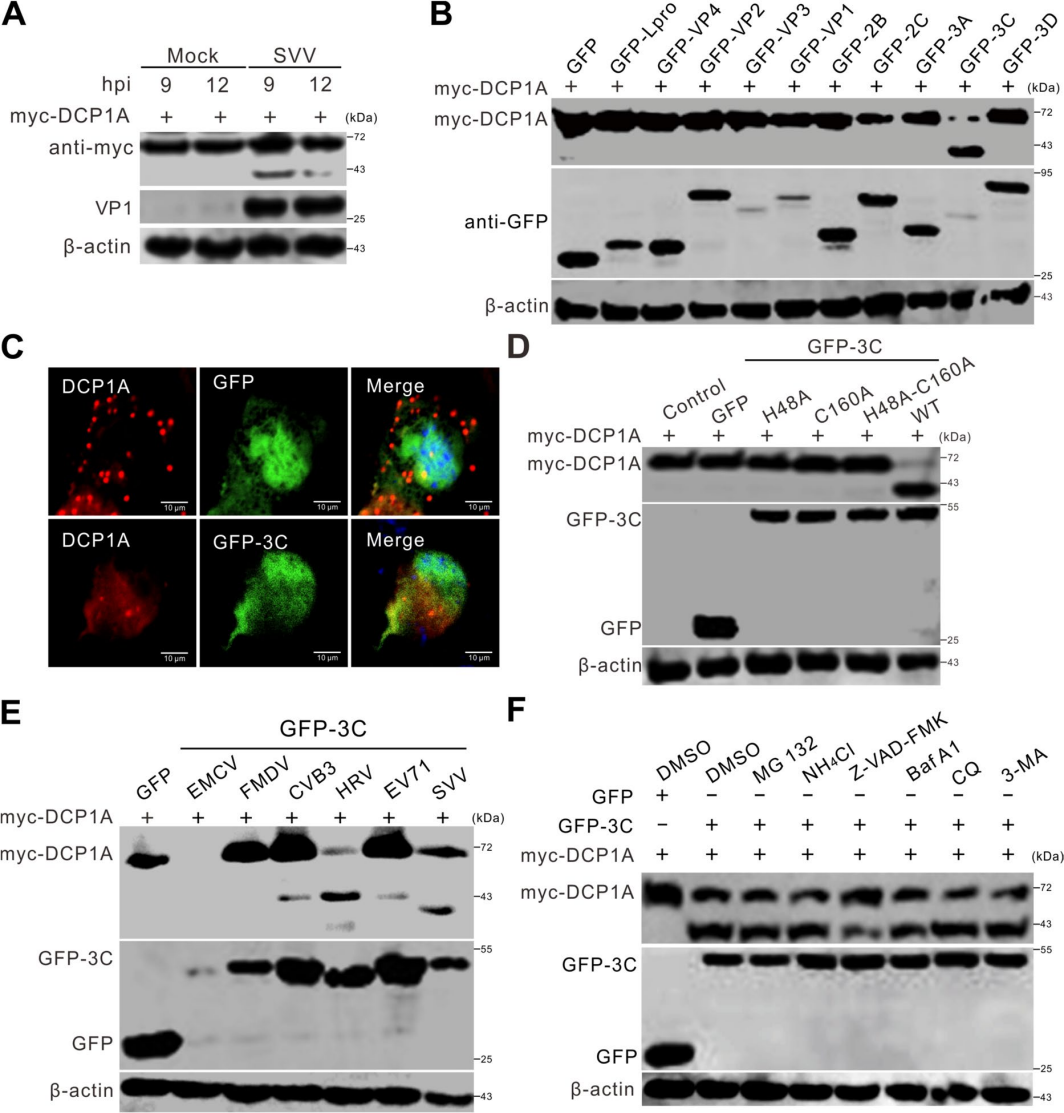
**SVV 3C**^**pro**^** cleaves DCP1A. A** BHK-21 cells seeded in 6-well plates were transfected with 1 μg myc-DCP1A for 24 h, followed by infection with SVV at an MOI of 5. At the indicated times, the cells were harvested and subjected to western blotting analysis with antibodies against myc, VP1 and β-actin. **B** BHK-21 cells cultured in 6-well plates were cotransfected with 2 μg of plasmids encoding GFP-tagged SVV proteins along with 1 μg of myc-DCP1A. At 24 hpt, the cells were harvested and subjected to western blotting analysis to determine the expression levels of myc-DCP1A. **C** BHK-21 cells seeded in 6-well plates were transfected with 2 μg of the GFP or GFP-3C plasmids. At 24 hpt, the cells were fixed, permeabilized, stained with antibodies against the DCP1A protein, and then examined via confocal microscopy. **D** BHK-21 cells seeded in 6-well plates were cotransfected with constructs encoding myc-DCP1A (1 μg) and GFP-tagged 3C or its mutant derivatives (GFP-3C^H48A^, GFP-3C^C160A^, and GFP-3C^H48A−C160A^) (2 μg). Western blotting analysis was performed to examine the expression levels of myc-DCP1A. **E** BHK-21 cells cultured in 6-well plates were cotransfected with 1 μg of myc-DCP1A and 2 μg of plasmids encoding GFP-tagged 3C proteins derived from encephalomyocarditis virus (EMCV), foot and mouth disease virus (FMDV), coxsackievirus B3 (CVB3), human rhinovirus (HRV), enterovirus 71 (EV71), and SVV. The cells were subjected to western blotting analysis to evaluate the expression levels of myc-DCP1A. **F** BHK-21 cells seeded in 6-well plates were cotransfected with 1 μg of myc-DCP1A and 2 μg of GFP-3C or GFP plasmids, followed by treatment with MG132 (10 μM), NH4Cl (10 mM), Z-VAD-FMK (50 μM), bafilomycin A1 (Baf A1, 200 nM), chloroquine (CQ, 40 μM), or 3-methyladenine (3-MA, 25 mM). The levels of myc-tagged DCP1A expression were subsequently evaluated via western blotting analysis.

Having confirmed that SVV infection cleaved endogenous DCP1A, we further investigated whether SVV infection could also cleave overexpressed DCP1A. To this end, BHK-21 cells were first transfected with myc-DCP1A for 24 h, followed by infection with SVV. As shown in Figure 3A, western blotting analysis indicated that SVV infection also led to the cleavage of overexpressed DCP1A (Figure 3A). To identify which viral protein contributes to the cleavage of DCP1A, we cotransfected SVV-encoded structural and nonstructural protein plasmids with myc-DCP1A. Western blotting revealed that only cells coexpressing 3C^pro^ and DCP1A exhibited a cleavage band identical to that observed during viral infection (Figure 3B). Confocal microscopy revealed that the overexpression of 3C^pro^ significantly reduced the number of endogenous DCP1A molecules with a punctate distribution (Figure 3C). The histidine at position 48 and the cysteine at position 160 of 3C^pro^ are key sites for its protease activity, and mutations at these sites, either singly or in combination, inhibit the proteolytic activity of 3C^pro^ [26]. To determine whether the protease activity of 3C is related to the cleavage of DCP1A, we constructed mutants with inactivated protease activity of 3C and cotransfected them with DCP1A. We observed that cells overexpressing 3C^H48A^, 3C^C160A^, and 3C^H48A−C160A^ continued to express DCP1A and did not yield a cleavage band (Figure 3D), indicating that 3C^pro^ catalytic sites are crucial for the protein’s ability to cleave DCP1A. To assess whether the function of 3C is consistent across *Picornaviridae*, we examined 3C^pro^ from encephalomyocarditis virus (EMCV), foot and mouth disease virus (FMDV), coxsackievirus B3 (CVB3), human rhinovirus (HRV), and enterovirus 71 (EV71). Interestingly, CVB3, HRV, and EV71 3C^pro^ also cleave DCP1A (Figure 3E). In contrast, SVV targets DCP1A for cleavage, resulting in a smaller product than those of CVB3, HRV, and EV71 (Figure 3E). To further evaluate the pathway involved in the cleavage of DCP1A by 3C^pro^, we cotransfected cells with 3C^pro^ and DCP1A and subsequently treated these cells with the proteasome inhibitor MG132, the caspase inhibitor Z-VAD-FMK, the lysosome inhibitor NH_4_Cl, or the autophagy inhibitors Baf A1, CQ, and 3-MA. Western blotting analysis revealed that none of the inhibitors prevented the formation of DCP1A cleavage bands. Therefore, 3C^pro^-mediated cleavage of DCP1A was not dependent on caspases, autophagy, or proteasomal pathways (Figure 3F). Collectively, these results demonstrated that SVV 3C^pro^ targeted DCP1A for cleavage.

### SVV 3C^pro^-mediated cleavage of DCP1A abolished its antiviral activity

**Figure 4:**
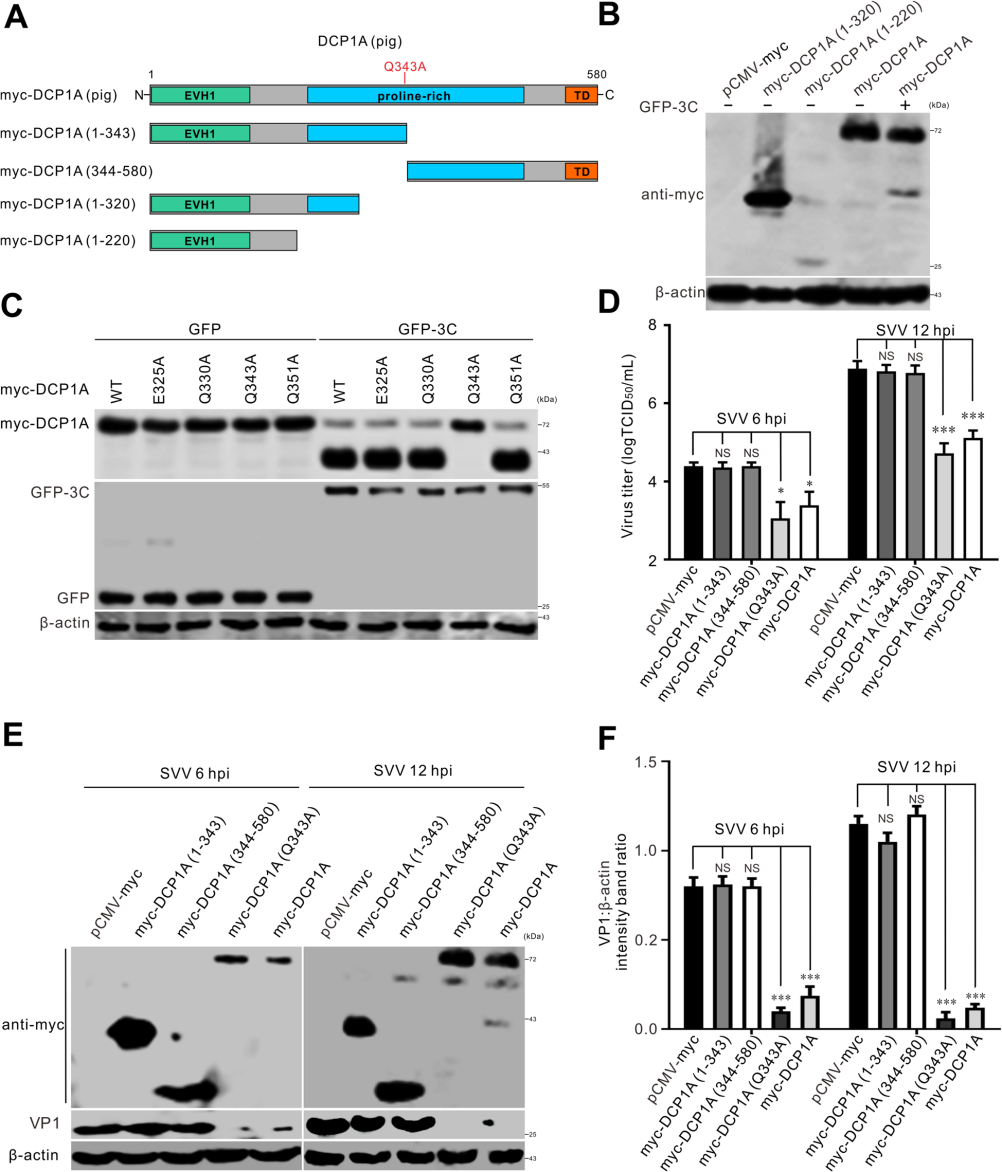
**The antiviral activity of cleaved DCP1A was abolished. A** A schematic representation illustrating the structural domains and truncated mutants of DCP1A proteins. **B**, **C** BHK-21 cells seeded in 6-well plates were cotransfected with 2 μg of GFP-3C and 1 μg of myc-tagged DCP1A, its truncated constructs, or point mutants. At 24 hpt, western blotting analysis was performed to determine the protein levels of GFP-3C. **D**, **E** BHK-21 cells plated in 6-well plates were first transfected with 1 μg of myc-tagged DCP1A or its truncation constructs, followed by SVV infection at 24 hpt. TCID_50_ and western blotting assays were used to evaluate the virus titers in the supernatants (**D**) and the expression of the VP1 protein (**E**). The data are presented as the means ± standard deviations (SDs) derived from three independent experiments. (NS, not significant; *P < 0.05; ***P < 0.001). **F** Quantitative analysis shows the levels of VP1 normalized against β-actin in **E**, as determined via Image J software. The data are presented as the means ± standard deviations (SDs) derived from three independent experiments. (NS, not significant; ***, *P* < 0.001).

To identify the DCP1A cleavage site mediated by 3C^pro^, we generated truncated DCP1A (1–320) and DCP1A (1–220) fragments on the basis of the molecular weights of the cleaved DCP1A fragments (Figure 4A). Western blotting assays revealed that the size of the cleaved N-terminal myc-DCP1A product was slightly greater than that of the DCP1A (1–320) mutant (Figure 4B), indicating that the potential cleavage site of DCP1A was located near the 320 amino acid position. The 3C protease from picornaviruses specifically recognizes and cleaves proteins with glutamine-glycine (Q-G) or glutamic acid–glutamine (E-Q) sequences [27]. Thus, we constructed four mutants based on known cleavage targets of 3C, DCP1A (E325A), DCP1A (Q330A), DCP1A (Q343A), and DCP1A (Q351A), and cotransfected them with SVV 3C to verify the latter’s cleavage sites. As shown in Figure 4C, only DCP1A (Q343A) was resistant to 3C cleavage, whereas DCP1A (E325A), DCP1A (Q330A), and DCP1A (Q351A) were cleaved. Hence, residue Q343 of DPC1A is the target site for the 3C protease. Having confirmed that DCP1A inhibited SVV replication, we further investigated whether the cleaved products retained antiviral activity. To this end, we transfected BHK-21 cells with the cleaved products DCP1A (1–343) and DCP1A (344–580) and a non-cleavable form of DCP1A (Q343A), and then infected them with SVV. As shown in Figures 4D–F, TCID_50_ and western blotting assays revealed that viral titers and VP1 protein levels at 6 and 12 hpi were significantly lower in cells transfected with DCP1A (Q343A) and DCP1A than in cells transfected with DCP1A (1–343), DCP1A (344–580), or the empty vector. Together, these findings suggest that 3C^pro^ cleaves DCP1A at Q343, resulting in two fragments, namely, DCP1A (1–343) and DCP1A (344–580), both of which have lost the antiviral activity of the full-length DCP1A.

### DCP1A degrades 3D viral RNA-dependent RNA polymerase

**Figure 5:**
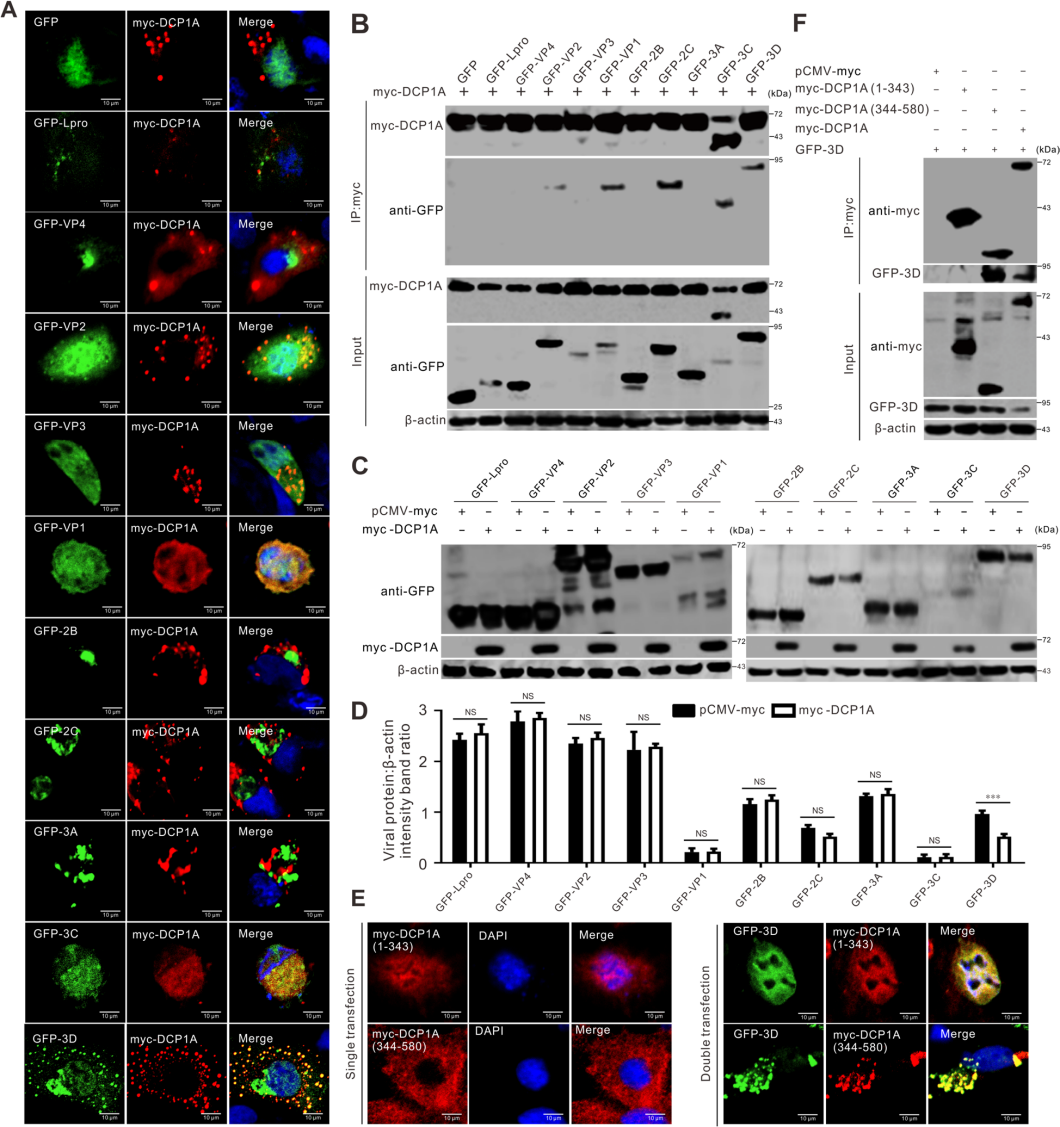
**DCP1A degrades 3D.** **A** BHK-21 cells cultured in 6-well plates were cotransfected with plasmids encoding GFP-tagged SVV proteins (2 μg) along with myc-tagged DCP1A (1 μg). At 24 hpt, the cells were fixed, permeabilized, stained with a myc-tagged antibody (red) and DAPI (blue), and then examined via confocal microscopy. **B** BHK-21 cells cultured in 6-well plates were cotransfected with plasmids encoding myc-DCP1A (1 μg) and GFP-tagged SVV proteins (2 μg). At 24 hpt, the cells were harvested and subjected to coimmunoprecipitation (co-IP) using anti-myc magnetic beads. Western blotting analysis was subsequently performed to examine the precipitated proteins. **C** BHK-21 cells in 6-well plates were cotransfected with 2 μg of myc vector or myc-DCP1A with 1 μg of GFP-tagged SVV protein-coding plasmids. Western blotting analysis was performed to evaluate the expression of the GFP-tagged viral proteins. **D** Quantitative analysis shows the levels of GFP-tagged viral proteins normalized against β-actin in **C**, as determined via Image J software. The data are presented as the means ± standard deviations (SDs) derived from three independent experiments. (NS, not significant; ***, *P* < 0.001). **E** BHK-21 cells cultured in 6-well plates were transfected with myc-tagged DCP1A truncations alone (1 μg) or cotransfected with GFP-3D (2 μg) and myc-tagged DCP1A truncations (1 μg). At 24 hpt, the cells were fixed, permeabilized, stained with a myc-tagged antibody (red) and DAPI (blue), and then examined via confocal microscopy. **F** BHK-21 cells in 6-well plates were cotransfected with 1 μg of myc vector or myc-tagged DCP1A, or its truncates with 2 μg of GFP-3D for 24 h. Co-IP assays coupled with western blotting analysis were performed to determine the interacting proteins.

To understand the potential mechanisms of DCP1A action, we investigated interactions between SVV proteins and DCP1A. BHK-21 cells were cotransfected with selected SVV proteins and DCP1A, and then subjected to laser confocal microscopy and co-IP assays. As shown in Figure 5A, GFP-tagged VP1, VP2, 3C, and 3D colocalized with myc-DCP1A, and the coexpression of VP2 or 3D with DCP1A resulted in a punctate distribution. The overexpression of 3C disrupted the punctate distribution of myc-tagged DCP1A, similar to the results observed in SVV-infected cells (Figure 1J) and endogenous DCP1A (Figure 3C), indicating that 3C abrogates the function of DCP1A. Co-IP assays confirmed that VP1, VP2, 2C, 3C, and 3D interact with DCP1A (Figure 5B). To further determine the effect of DCP1A on each viral protein, we cotransfected viral proteins with DCP1A or an empty vector. Western blotting analysis revealed that DCP1A specifically degrades 3D proteins (Figures 5C and D). We also examined the colocalizations and interactions between 3D and cleaved DCP1A products. Only DCP1A (344–580) resulted in the colocalization of full-length DCP1A and 3D (Figure 5E). Consistent with these results, DCP1A (344–580) was responsible for the interaction of full-length DCP1A with 3D (Figure 5F). Notably, both DCP1A (1–343) and DCP1A (344–580) abrogated the ability to induce 3D degradation as the full-length DCP1A does (Figure 5F). Together, these results reveal that DCP1A interacts with and selectively degrades 3D viral RNA-dependent RNA polymerase.

### DCP1A targets 3D for OPTN-mediated autophagic degradation

**Figure 6:**
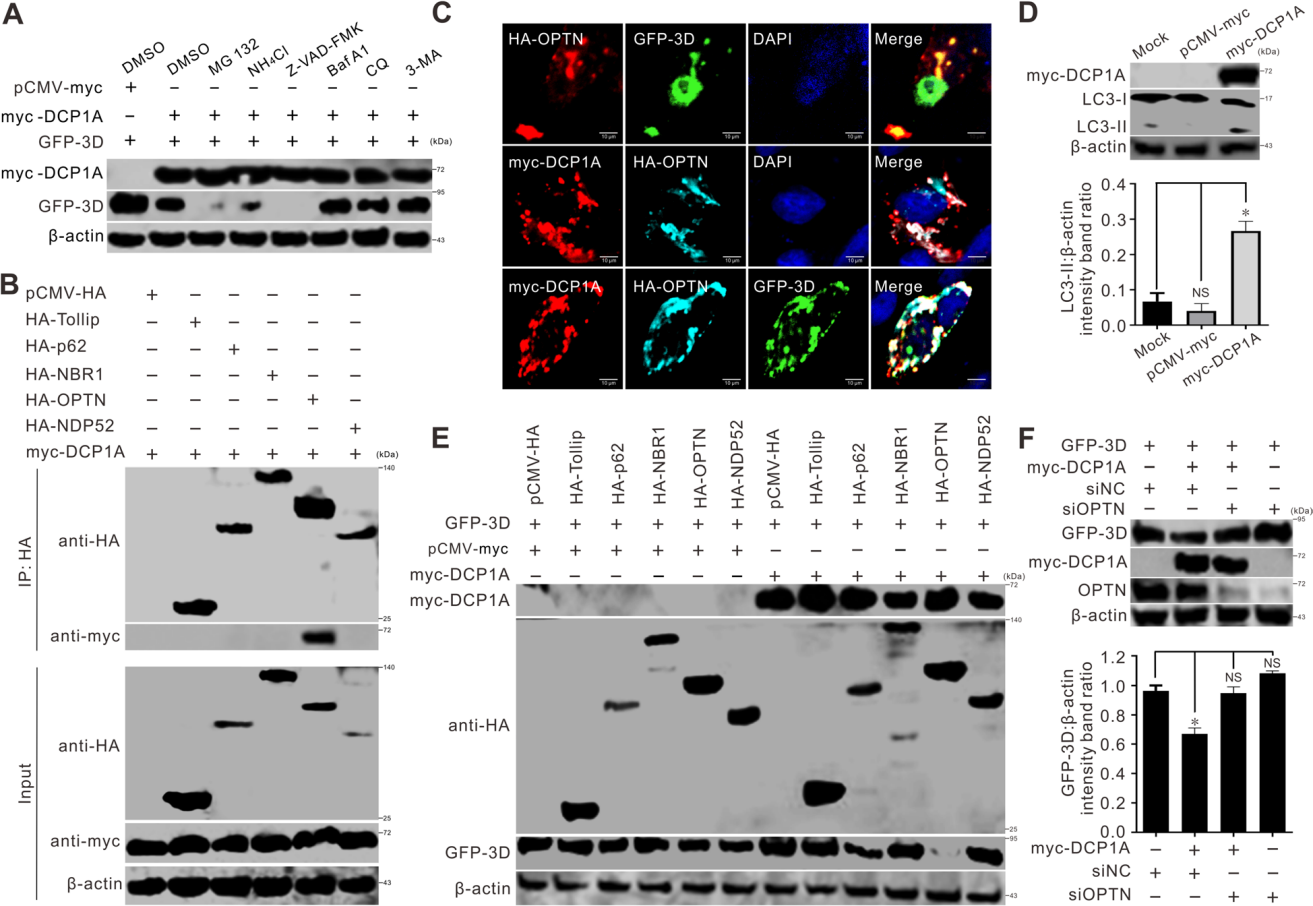
**DCP1A targets 3D for OPTN-mediated autophagic degradation. A** BHK-21 cells in 6-well plates were cotransfected with 2 μg of myc-DCP1A and 1 μg of GFP-3D for 24 h. After treatment with the indicated inhibitors for 12 h, western blotting was performed to assess the GFP-3D expression. **B** BHK-21 cells in 6-well plates were cotransfected with 1 μg of myc-DCP1A and 2 μg of the HA-tagged autophagy receptors. At 24 hpt, a co-IP assay was performed to analyze the precipitated proteins. **C** BHK-21 cells in 6-well plates were either double-transfected with HA-OPTN and GFP-3D or myc-DCP1A or triple-transfected with HA-OPTN, GFP-3D and myc-DCP1A for 24 h (1 μg of each plasmid). The cells were fixed, permeabilized, stained with antibodies and DAPI, and examined via confocal microscopy. **D** BHK-21 cells in 6-well plates were transfected with 1 μg of myc-DCP1A for 24 h. Western blotting was performed to determine the expression of LC3-I and LC3-II. Quantitative analysis of the levels of LC3-II normalized to those of β-actin via Image J software. The data are presented as the means ± standard deviations (SDs) derived from three independent experiments. (NS, not significant; *, *P* < 0.05). **E** BHK-21 cells in 6-well plates were triple-transfected with GFP-3D, myc-DCP1A and the HA-tagged autophagy receptors (1 μg of each plasmid). At 24 hpt, western blotting was performed to determine the GFP-3D expression. **F** BHK-21 cells in 6-well plates were first transfected with siRNAs (20 pmol) for 36 h, followed by cotransfection with 2 μg of GFP-3D and 1 μg of myc-DCP1A. Western blotting was used to evaluate the expression of OPTN and GFP-3D. Quantitative analysis of the levels of GFP-3D normalized to those of β-actin via Image J software. The data are presented as the means ± standard deviations (SDs) derived from three independent experiments. (NS, not significant; *P < 0.05).

The main pathways for intracellular protein degradation are the ubiquitin–proteasome, autophagy, lysosome, and caspase-dependent pathways. To determine the pathway by which DCP1A deploys 3D degradation, BHK-21 cells were cotransfected with DCP1A and 3D, followed by treatment with a proteasome inhibitor (MG132), a lysosome inhibitor (NH_4_Cl), the autophagy inhibitors Baf A1, CQ, and 3-MA; a caspase inhibitor (Z-VAD-FMK); or a solvent for inhibitors (dimethyl sulfoxide, DMSO). Western blotting assays revealed that 3D expression was significantly greater in the autophagy inhibitor groups than in the other inhibitor groups, suggesting that the DCP1A-induced 3D degradation was autophagy-dependent (Figure 6A). A series of studies verified that autophagy receptors act as intermediaries, recognizing the targeted substrate for autophagy and facilitating the selective autophagy process [24]. Thus, we used co-IP and confocal microscopy assays to assess which autophagy receptor was involved in the DCP1A-induced degradation of 3D. Among the tested receptors, only OPTN interacted (Figure 6B) and colocalized (Figure 6C) with DCP1A. Intriguingly, the colocalization of OPTN and 3D was strengthened in the presence of DCP1A (Figure 6C). On the basis of these findings, we hypothesized that DCP1A recruits OPTN to target 3D for autophagic degradation. To test this hypothesis, we first examined whether DCP1A could induce autophagy. The conversion of LC3-I to LC3-II is a marker of autophagy, and the amount of LC3-II generated is proportional to the number of autophagosomes [28]. Therefore, we examined the ratio of LC3-I to LC3-II in the presence of DCP1A. As shown in Figure 6D, the levels of LC3-II in cells transfected with DCP1A were significantly greater than those in cells transfected with an empty vector or mock control, indicating that DCP1A induces autophagy. Second, to assess the effects of OPTN and DCP1A on 3D, we cotransfected cells with plasmids encoding DCP1A, 3D, and different autophagy receptors. The results indicated that the 3D protein levels significantly decreased only in cells coexpressing DCP1A, 3D, or OPTN (Figure 6E). We further knocked down OPTN via specific siRNAs to confirm the role of OPTN in mediating 3D autophagic degradation. As shown in Figure 6F, OPTN silencing restored DCP1A-mediated 3D degradation. Collectively, these results indicate that OPTN-mediated autophagy is responsible for DCP1A-induced 3D degradation.

## Discussion

As the predominant group of innate antiviral cytokines, IFNs act by inducing the expression of ISGs. The evolutionary arms race between viruses and hosts has resulted in viruses evolving mechanisms to evade host immune surveillance, including the modulation of ISGs [29, 30]. One major ISG with broad-spectrum antiviral activity is DCP1A [21]. Our study revealed that DCP1A recruits the autophagy receptor OPTN to degrade the viral 3D protein and inhibit viral replication. In response, the viral 3C protease cleaves and degrades DCP1A to block its antiviral function.

Our results revealed that SVV infection induced DCP1A cleavage, caused DCP1A degradation, and disrupted the localization of DCP1A (Figures 1A–J). SVV 3C^pro^ is attributed to the cleavage of DCP1A, which is dependent on its protease activity (Figures 3A–D). Interestingly, nsp5 of PDCoV, alphacoronaviruses (PEDV, HCoV-229E, HCoV-NL63, SADS-CoV), and betacoronaviruses (HCoV-OC43, HCoV-HKU1, SARS-CoV, SARS-CoV-2, MERS-CoV), is a 3C-like protease that cleaves DCP1A at residue Q343 [11, 19, 20]. Our results indicated that SVV 3C^pro^ also cleaves DCP1A at residue Q343 (Figures 4A–C). Other members of *Picornaviridae*, including CVB3, HRV, and EV71, also cleave DCP1A, but the cleavage site is different from that of SVV (Figure 3E). These results suggest that viruses have evolved similar strategies to antagonize host intrinsic antiviral factors. DCP1A was predominantly localized in the cytoplasm and exhibited a punctate distribution. Upon SVV infection, the number of punctate aggregations disappeared, and diffuse cytoplasmic staining was observed (Figure 1J). Further study revealed that SVV 3C^pro^ was responsible for disrupting the punctate distribution of DCP1A (Figures 3C and 5A). Significantly, the cleaved DCP1A products, namely, DCP1A (1–343) and DCP1A (344–580), were located in the nucleus and cytoplasm, respectively, without a punctate distribution (Figure 5E). The DCP1A protein is a crucial marker of the P body. Poliovirus infection disrupts P bodies, which is manifested by a reduction in the number of P-body foci within infected cells, and viral 3C^pro^ promotes P-body cleavage [18]. CVB3 infection also elicits this response, leading to a comparable loss of P-bodies [18]. After cleavage, the degradation of 3D, induced by DCP1A, was terminated (Figure 5F). Consistent with these results, the cleaved DCP1A products lost their capacity to suppress SVV replication (Figures 4D–F). These findings suggested that SVV 3C^pro^ cleaves DCP1A to suppress its ability to defend against SVV infection, as reflected by the fact that the degradation of 3D was restrained following the cleavage of DCP1A (Figure 5F).

Responsible for cleaving the SVV polyprotein precursor, 3C^pro^ exerts a multifaceted effect on the host cell [31, 32], disrupting signal transduction, nuclear transport, transcription, and translation [32]. This interference of host cell functions at multiple levels dampens the host immune response and enhances viral replication, contributing significantly to SVV pathogenicity [32]. For example, SVV 3C^pro^ extensively inhibits type I IFN signaling in hosts [33]. In porcine cells infected with SVV, type I IFN is activated by RIG-I signaling [34]. The latter is crucial in host detection and defence against viral RNA. The key molecules in the RIG-I pathway include RIG-I, MAVS, TBK1, TRAF3, TRIF, TANK, and IRF3/7 [34]. SVV 3C^pro^ suppresses this signaling pathway through the following mechanisms: degradation of RIG-I or IRF3/7 [35]; cleavage of MAVS, TRIF, TANK, or NF-κB [33, 36]; and inhibition of protein interactions, such as those of MAVS and RIG-I, through increased lactate production [37]. Furthermore, 3C^pro^ can inhibit RIG-I, TBK1, and TRAF3 ubiquitination [38]. In this study, we revealed that 3C degraded and cleaved DCP1A at residue Q343, providing new details on its ability to regulate host innate immunity.

Host-cell synthesis of ISGs and viral interference with their function are clear examples of host-virus coevolution [39]. The early and middle phases (but not the late phase) of SVV infection led to strong upregulation of Mx1 and interferon-stimulating gene 15 (ISG15) in host cells. Mx1 inhibits SVV replication in PK-15 cells through interactions with the capsid proteins VP1, VP2, and VP3 [40]. IFITM1 and IFITM2 inhibit SVV replication by promoting the expression of RIG-I and associated IFNs [41]. Additionally, IFIT3 mediates the antiviral response of type I IFNs by targeting SVV entry, assembly, and release pathways [42]. In response, SVV infection increases the expression of migration inhibitory factors (MIFs), and these MIFs then suppress the expression of ISG15 and ISG56 [43]. Another target of SVV is the host factor suppressor of cytokine signaling 1, which is upregulated to inhibit ISG56, ISG54, and PKR production [44]. Our study contributes to the current understanding of host-viral interactions, showing that DCP1A inhibits SVV replication by targeting the viral 3D for autophagic degradation, whereas SVV exploits its 3C^pro^ to cleave DCP1A.

Another major finding in this study is that DCP1A recruits OPTN to mediate 3D degradation. OPTN is a multifunctional protein that maintains the integrity of the Golgi apparatus [45, 46], negatively modulates NF-κB signaling [47], and regulates autophagy [48], specifically xenophagy. Xenophagy targets invading bacteria, viruses, and other pathogens for degradation, using autophagy receptors for pathogen recognition [49]. These receptors harbor a ubiquitin-binding domain (UBD) that binds to ubiquitinated intracellular cargo and a MAP1LC3/LC3 (microtubule-associated protein 1 light chain 3)-interacting region (LIR) that links the cargo to autophagosomes [49]. OPTN is among the known autophagy receptors involved in selective cargo uptake [50]. In addition to cargo recognition, OPTN is associated with autophagosome formation/maturation and lysosomal quality control [48]. Moreover, OPTN itself can be degraded through the autophagic process [51].

The versatile nature of OPTN is also apparent during viral infections. Phosphorylated OPTN targets the tegument protein VP16 and the fusion glycoprotein gB of herpes simplex virus type 1 (HSV-1) for autophagy, preventing virus-induced neurodegeneration [28, 52]. During HSV-1 infection, PLK1 regulates TBK1 to phosphorylate and activate OPTN [53]. OPTN is also upregulated in infectious bursal disease virus to prevent K63-linked ubiquitination of VP1, inhibiting the latter's polymerase activity [54]. While SVV deploys 3C^pro^ to cleave OPTN and impairs selective autophagy and type I IFN signaling [24], we observed that OPTN is also involved in SVV protein degradation. DCP1A can induce autophagy, as evidenced by the increased expression of LC3-II in DCP1A-transfected cells. The autophagy receptor OPTN was found to interact with DCP1A and colocalize with DCP1A and 3D. Moreover, OPTN degrades only 3D in the presence of DCP1A. These results suggest that DCP1A recruits OPTN to induce the autophagic degradation of 3D, which was further confirmed by the restoration of 3D protein levels upon OPTN knockdown. Therefore, we can conclude that DCP1A exerts its antiviral function through OPTN-mediated autophagic degradation of the 3D protein, possibly one of the mechanisms by which DCP1A functions as an antiviral factor, and there are still other questions to be further explored in this study, i.e., multiple viral proteins were found to interact with DCP1A, raising the question of whether other viral proteins also contribute to its antiviral function. We will strive to address these issues in future research.

In conclusion, this study demonstrated that DCP1A exerts an antiviral effect on SVV replication by targeting 3D viral RNA-dependent RNA polymerase for OPTN-mediated autophagic degradation. To counteract this effect, SVV 3C cleaves DCP1A at Q343, resulting in cleaved fragments without antiviral potency. These findings elucidate the molecular mechanism underlying the SVV response to antiviral defences, enhancing our understanding of host-virus dynamics.

## Data Availability

All the data generated or analysed during this study are included in this published article.
